# 462. Red Cell Exchange Transfusion for Treatment of Babesiosis

**DOI:** 10.1093/ofid/ofaf695.154

**Published:** 2026-01-11

**Authors:** David E Leaf, Audrey E Monson, Julie-Alexia Dias, Peter J Krause

**Affiliations:** Brigham and Women's Hospital, Boston, Massachusetts; Brigham and Women's Hospital, Boston, Massachusetts; Harvard T.H. Chan School of Public Health, Boston, Massachusetts; Yale School of Public Health and Yale School of Medicine, Sudbury, Massachusetts

## Abstract

**Background:**

Red cell exchange transfusion (ET) has been used as an adjunctive treatment for severe illness from babesiosis, particularly in patients with high parasitemia, acute organ injury, or severe hemolysis. However, data supporting the efficacy of ET on improving clinical outcomes are lacking.

**Figure:**
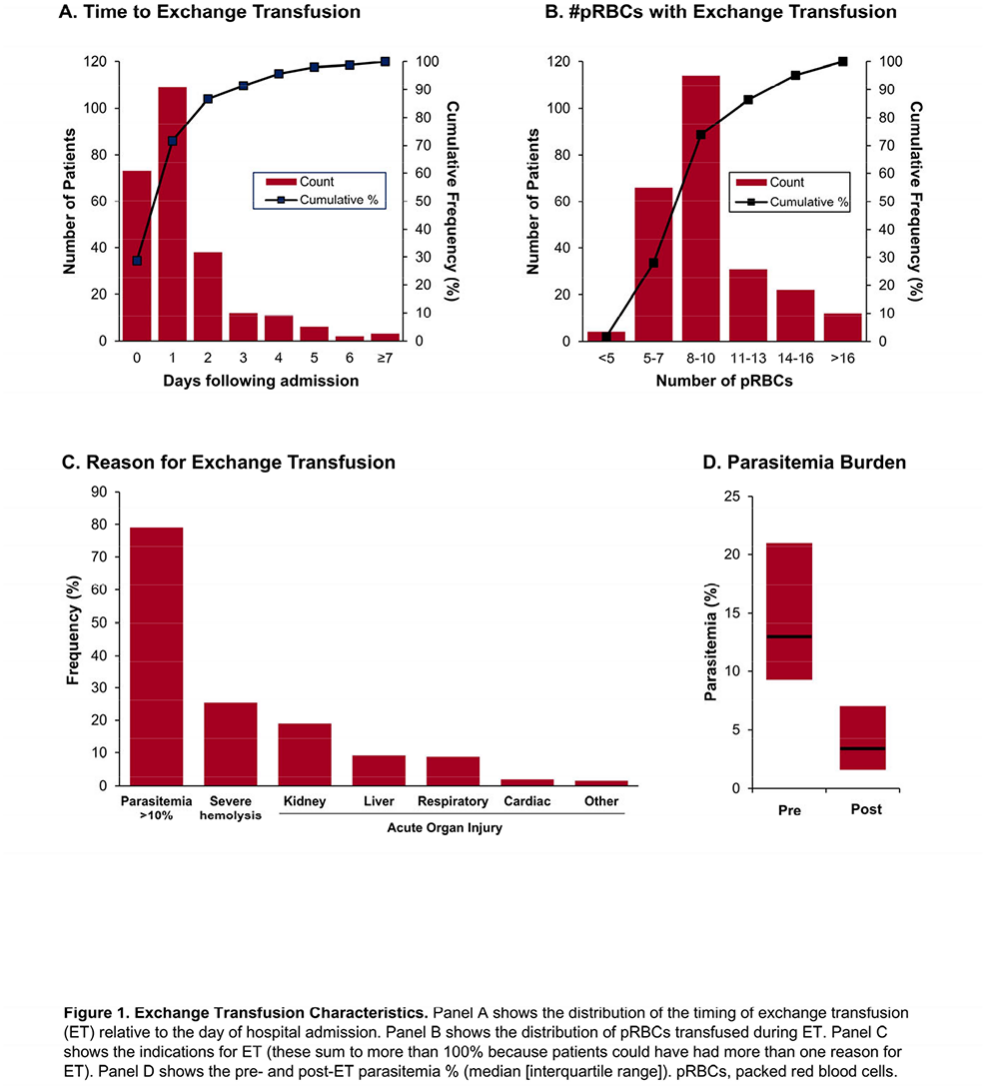

**Figure:**
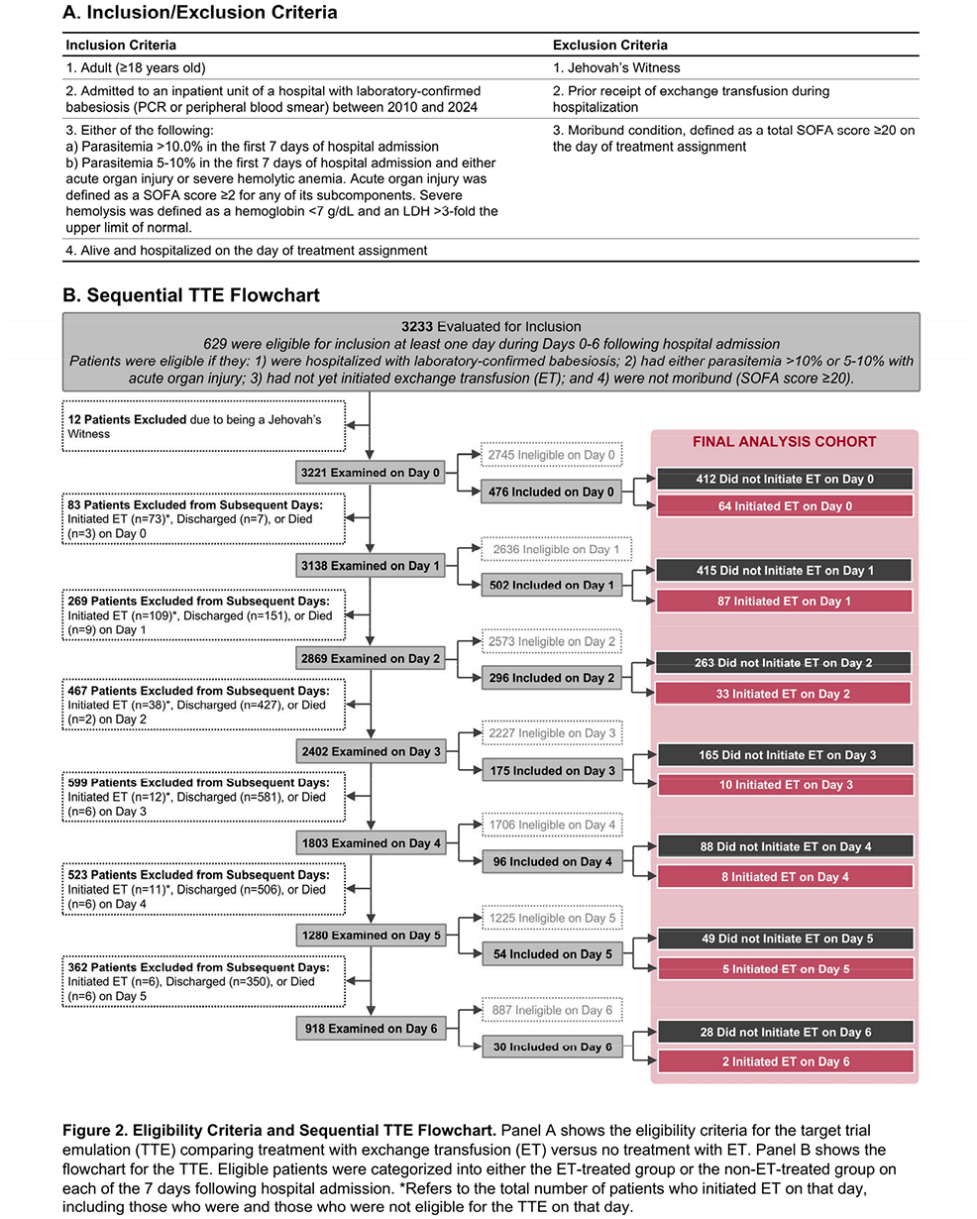

**Methods:**

We performed a multicenter cohort study of 3,233 consecutive adults hospitalized with babesiosis at 84 hospitals across the northeastern US from 2010 to 2024. Data on demographics, comorbidities, vital signs, physiologic parameters, labs, treatments, and outcomes were collected by detailed chart review. Patients were eligible for this analysis if they had >10% parasitemia or 5-10% parasitemia with either acute organ injury or severe hemolytic anemia. To minimize the potential for indication- and immortal-time biases, we used a sequential target trial emulation (TTE) framework. To do so, we categorized eligible patients according to whether they did or did not initiate ET on each of the first 7 days of hospital admission. The primary outcome was a composite of in-hospital death or 30-day readmission. We used a logistic regression model with inverse probability of treatment weighting (IPTW) to adjust for confounding.

**Figure:**
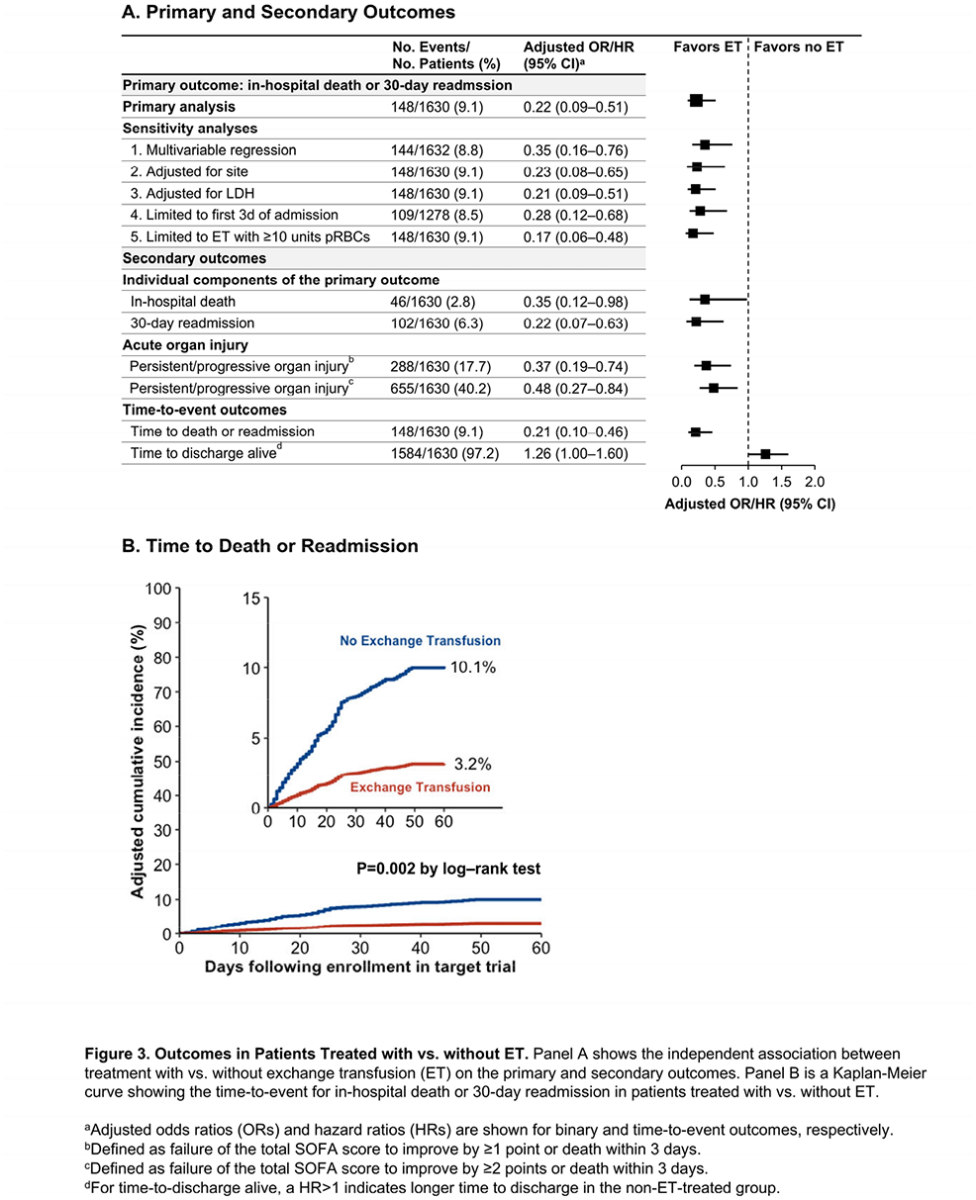

**Figure:**
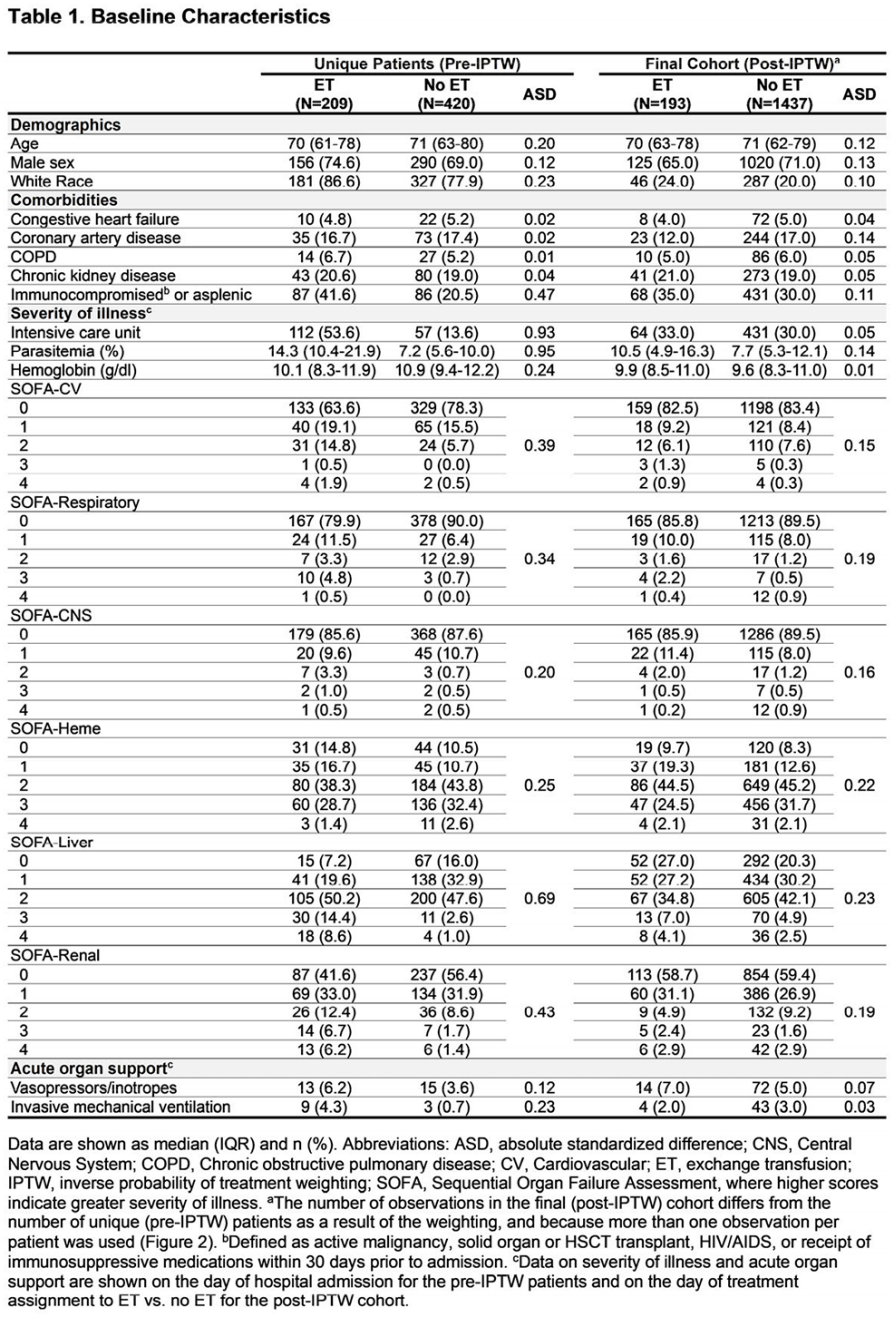

**Results:**

A total of 254 of 3,233 patients (7.9%) received ET, 98.4% of whom initiated it in the first 7 days of hospitalization (Figure 1). Among 629 unique patients eligible for inclusion in at least 1 of the 7 sequential TTEs, 209 (33.2%) received ET (Figure 2). ET-treated patients had greater severity-of-illness compared to non-ET-treated patients; however, these characteristics were well-balanced after applying IPTW (Table 1). Overall, 60 patients (9.5%) had a primary outcome event. In the primary analysis, patients who received ET had a lower odds of developing the primary outcome compared to those who did not (odds ratio, 0.22 [95% CI, 0.09–0.51]; Figure 3). Results were consistent across a number of sensitivity and secondary analyses (Figure 3A) and in a time-to-event analysis (Figure 3B).

**Conclusion:**

In this multicenter cohort study of severely ill hospitalized adults with babesiosis, the risk of in-hospital death or 30-day readmission was nearly 5-fold lower in those treated with vs. without ET. These data support the use of ET for patients with severe illness from babesiosis.

**Disclosures:**

All Authors: No reported disclosures

